# COVID-19 and the Political Economy of Shared Adjustment

**DOI:** 10.1007/s10272-021-0999-0

**Published:** 2021-10-05

**Authors:** Ralf Boscheck

**Affiliations:** IMD SWITZERLAND, Ch. de Bellerive 23, 1001 Lausanne, Switzerland

## Abstract

By April 2021, the COVID-19 crisis in Europe had reached a magnitude that, in the eyes of some observers, either deepened lingering divides and threatened the EU’s very existence, or, conversely, forced the Union to address the fundamental flaws of its euro area and provided an opportunity to reboot. From the outset, the EU had to confront fundamental challenges that require coordination; however, decentralised coordination is best as it improves the quality of policy, economic efficiency and civic virtues. While some argue for a debt union to provide the answer to the EU’s call for shared adjustment, a solution should rather be sought in economic reform, accountability and enforcement of constitutional commitments.

In 2020, the COVID-19 crisis resulted in the largest global economic contraction since 1946. For 2021-22, most macroeconomic experts predicted the recovery would most likely be K-shaped, with some economies and parts of society recuperating faster and others facing a more lengthy and difficult resurgence. And yet, behind this very general and therefore innocuous statement was a considerable disagreement over adequate policy responses, their short-term consequences and long-term side effects. What is more, some enduring policy tenets appeared to have lost credibility at a time when countries sought direction on how to shoulder the fiscal burden or establish the legitimacy of adjustments to block any further epidemic and economic contagion.

By April 2021, the crisis in Europe had reached a magnitude that, in the eyes of some observers, either deepened lingering divides and threatened the EU's very existence, or, conversely, forced the Union to address the fundamental flaws of its euro area and provided an opportunity to reboot. This article provides a critical perspective on the EU's ability to manage a shared adjustment to the coronavirus pandemic.

**Ralf Boscheck**, Universidad Adolfo Ibáñez, Santiago de Chile, Chile; International Institute for Management Development, Lausanne, Switzerland.

## EU governance: Fundamentals, post-2008 and COVID-19

In the early 1950s, Robert Schuman and Jean Monnet, the founding fathers of the EU, tried to lay the institutional foundation for economic prosperity in order to stabilise a war-ridden, divided continent. Ever since, the growing diversity of economic capacities and policy preferences at the national and regional levels have presented the single most important challenge to the pursuit of the EU's core objectives: market creation, policy coordination and cohesion.

Europe is but a label. Any "normal" economic state covers nations with widely different GDP growth rates, sectoral compositions, unemployment rates, labour productivity and average manufacturing wages. While disparities among European nations are pronounced, regional differences within them are becoming economically and politically ever more important. Among the Union's 250 regions, GDP per capita is typically three times higher in the ten wealthiest countries than in the ten at the bottom of the scale. Differences in skills and infrastructures explain patterns of economic activity and rising income polarisation – the coexistence of regional growth magnets and poverty traps. Enlargement has added to this and the complexity of decision making.

Past efforts to speed up policymaking and enhance the Union's management role have largely failed. Early on, France's threat to withdraw from the Council allowed it to retain national veto powers on all matters of "vital national interest" until the adoption of the Single European Act in 1986. Thereafter, consultation and cooperation procedures were to centralise policymaking power in the Commission, and qualified majorities were to replace unanimity in taking substantive policy decisions. But many of the resulting policies were simply not executed. On several occasions following the enlargement of the EU, larger member countries demanded a re-weighing of EU Council votes to avoid minorities blocking decisions that often required a qualified majority. But such adjustments typically came at a high political cost and lost operational efficiency. Clearly, political representation within the EU is a major cause for concern; but so are member states' strategies for setting policy agendas, shaping legislation or obstructing implementation.

Under these conditions, and for many, the creation of the European Monetary System (EMS) must be considered a great achievement. But the benefits of the EMS – deeper capital markets, better risk allocation, enhanced contestability and trade creation – are not free. They call for the abolition of national monetary policy as a means of adjustment; the realisation that Europe, as a non-optimal currency area (Mundell, 1961), requires strong fiscal constraints; and the recognition that, with national monetary and fiscal policy adjustment severely limited, a country's ability to confront economic cycles reflects the flexibility of its labour markets and social policy provisions.

Clearly, unsynchronised business cycles, inflexible product and factor markets and little practice of fiscal solidarity make Europe susceptible to asymmetric shocks. Fiscal rules, like those enshrined in the 1997 Stability and Growth Pact (SGP) were to lessen pressures for more expansive monetary policies and the risk of crowding out private sector or, in a common financial market, smaller country borrowers. In addition, fiscal restraints were to limit national discretion or function as a form of self-restraint to curb domestic rent-seeking behaviour. But this idea raises two concerns. First, painful fiscal consolidation may attain the targets in the short term but may be difficult to sustain thereafter. Second, simple rules, such as a budget deficit limit of 3% and a debt ratio of a maximum of 60% of GDP may be considered too rigid to adjust to unexpected conditions. And so, gaming and political concessions were to be expected and became rampant in the precursor and aftermath of the 2008 economic crisis.

As early as 2005, the EU, finding it difficult to punish large member countries for violating their fiscal commitments, proposed the acceptance of a breach of the 3% deficit limit given exceptional circumstances. At that point, critics pointed out that, given the EU impending demographic challenge,[1] the debt and deficit limits should be made more restrictive, rather than loosened, to ensure intergenerational justice and prevent current generations living at the expense of future ones.

As a result, and in response to the European debt crisis of 2009, the EU attempted to tighten its grip, introducing greater macroeconomic surveillance and committing member countries to individual medium-term budgetary objectives based on the sustainable average limit for the country's structural deficit. This time, Brussels was criticised for effectively imposing an austerity rule that presumably dragged Greece, Italy, Portugal and Spain into a double-dip recession.[2] Only a few considered that, in the absence of viable national monetary and fiscal policy levers, a country's ability to confront economic shocks essentially reflects the accommodative capacities of its labour markets and social provisions. What is more, since the beginning of the decade, Northern European countries, like Germany, the Netherlands and Denmark, had set out to systematically restructure individual and collective labour laws and lower real unit labour costs. As a result, the growing asymmetry in social models between Europe's "frugal" north and its more "cozy" south and the incompatibility of this with the Economic and Monetary Union (EMU) became patently apparent (Hemerijck and Vandenbroucke, 2012). And yet, the EU Commission, under Jean-Claude Juncker, again gave in and set out to reinterpret SGP standards more leniently, in effect, tolerating the deterioration of structural budget balances. At the same time, the European Central Bank (ECB) began to guarantee government bonds of Southern European countries.

A decade later, COVID-19 destroyed incalculable value: lives, families and social structures, difficult to replace human, physical and financial capital, as well as consumer and investor confidence. EU countries have suffered an estimated reduction in annual GDP growth of up to three percentage points per month of lockdown (European Commission, 2020b). In May 2020, the EU Commission triggered the escape clause of the SGP, removing deficit limits to encourage member countries to increase public spending and alleviate the impact of the pandemic. In July 2020, the European Council approved a €750 billion Next Generation EU (NGEU) plan to ensure that the recov-

1 With an average EU fertility rate of 1.48 and a mean increase in life-expectancy of around 4.5 years since the 1960s, Europe's working population will have shrunk by almost 20.6 million in 2030, but it will have to shoulder an increased dependency ratio of then 66%. In addition, ageing will not only halve Europe's GDP growth potential from currently 2%-2.25% to 1.25% in 2040, but the demographic profile of their electorate may make it impossible for a range of EU states to reform their unfunded pay-as-you-go pension systems.

2 For a critical review of this argument, see Monastiriotis (2013).

ery is “sustainable, even, inclusive and fair for all Member States” (European Commission, 2020a). The fund is to be financed through borrowing by the EU, which will service the debt out of its (to be created) own tax revenues. Some €47.5 billion of the fund will be disbursed up to 2022, based on GDP losses, the level of youth unemployment and the relative prosperity of member states. The Recovery and Resilience Facility (RRF) amounts to €672.5 billion, made up of grants (€312.5 billion) and loans (€360 billion). The fund is not intended to cushion adjustment but should be allocated to member states in support of activities related to digital transformation, sustainability and the resilience of national economies.

Expectedly, the “frugal” countries within the Council, in particular Austria, Denmark, the Netherlands and Sweden, insisted on an increased loan portion and stronger Council controls over the approval and use of funds in the hands of the key recipients – France, Italy, Spain, Greece and Portugal. As regards the latter four, the EU Commission forecasted their average GDP levels to be driven back below their respective 2007 pre-crisis level, which, in the context of the SGP’s medium-term objectives, would require a massive fiscal consolidation. For some observers, it was “clear that a return to austerity policies would not be sustainable economically, socially or politically” (Truger, 2020). Others were wondering about the cost of borrowing, the relevance of fiscal rules and the political sustainability of the European Union (see for example, Gros, 2020a; for an early discussion see Boscheck, 2006). In the ensuing debate, contending views were displaying different levels of economic reasoning and bravado.

## Fiscal rules, zero interest rates and the “New Learning”

Keynes (1936, 383) argued that “(i)deas of economists and political philosophers are more powerful than is commonly understood. Indeed, the world is ruled by little else”. Nearly every day, new economic theories or adaptations of old ones are coming out of authoritative think tanks and hit the relevant policy communities. Impacts are not always predictable. Suddenly, some long-held policy dogmas appear to have lost force and the very institutions that had promoted them seem to be advocating the exact opposite or at least a strongly modified or fine-tuned approach. Suddenly, the IMF is no longer guided by the limits of the Washington Consensus and the OECD promotes the “rejection of standard numerical objectives and fiscal rules to allow economies to return to near normal levels” (Boone, as cited in Milenio, 2021). In economics, this is often called the “New Learning.” It serves as a reminder that persuasiveness in policy advice at times is not backed by irrefutable evidence, but by economists deploying rhetoric to suspend disbelief, cogently present their case and close the debate (Hood, 1993).

In some sense, this is what happened in March 2016. The authoritative Swiss *Neue Zürcher Zeitung* (2016) had just ridiculed the recent positions taken by the ECB and the US Fed on the needed extension of quantitative easing as “increasing the dose of a failing medicine”, as Harvard economist Summers (2014, 2016) popularised his interpretation of the situation.

Since 2008, the US, the EU and Japan had increased their debt-to-GDP ratios substantially. But for Summers and given his understanding of the reasons for prevailing low interest rates, this was not concerning. To explain the status, Summers invoked Hanson’s (1934) notion of “secular stagnation” and Wicksell’s (1898) idea of a “natural interest rate” to explain a balancing of savings, investments and full employment at rather low levels of growth. Summers provided some intuition to reason the excess supply of money relative to demand and found support in a Bank of England Working Paper arguing that the natural rate of interest for the OECD world had indeed dropped by 4.5% over the last 30 years (Rachel and Smith, 2015). Based on that, he argued that “monetary policy cannot address secular stagnation because the natural rate of interest is simply too low…but fiscal expansion raises demand. … It is true that an expansionary fiscal policy would increase deficits, and many worry that running larger deficits would place larger burdens on later generations who will already face the challenge of an ageing society. But those future generations will be better off owing lots of money in long-term bonds at low rates in a currency they can print than they would be inheriting a vast deferred maintenance liability” (Summers, 2016, 7).

Since then, several outstanding economists have endorsed similar positions. Krugman (2020) proposed a permanent stimulus of 2% of GDP with no tax increase, raising the US debt-to-GDP ratio close to 200% which, at current low levels of interest below growth rates, he deemed sustainable. Similarly, Blanchard (2019, 1197), in his presidential address at the American Economic Association, argued that “[i]f the future is like the past,… debt rollovers, that is the issuance of debt without a later tax increase, may well be feasible. Put bluntly, public debt may have no fiscal cost.” But he also cautioned that investors “requiring a risk premium, would increase the fiscal burden and make debt effectively more risky”; at any rate, Blanchard (2019, 1197) stressed that he did not “argue for more public debt…[but] to have a richer discussion of the costs of debt and of fiscal policy than is currently the case.”

In that case, however, some important qualifications should be added to the discussion (Gros, 2020b). For one, current and past growth levels should not be taken for granted; put differently, the future may not be like the past. If post-pandemic growth rates and inflation will be lower than in previous years, precisely due to the ineffectiveness of monetary policy, high deficits and debt levels will not be sustainable. Next, and in that context, "the coronavirus crisis is already the second 'once-in-a-century' crisis to hit us in the space of one decade" (Gros, 2020b, 282). With generally increased uncertainty, this would call for precaution and a limited increase in debt levels. Third, the pandemic affects sectors differently. Quite a few important implications arise from this: Clearly, any aggregate stimulus will be less effective than more focused interventions; fiscal multipliers will be higher as economies recover; and the argument that austerity is self-defeating cannot be sustained if frugality today permits a more effective fiscal boost tomorrow. At the same time, focused interventions amount to constituency support and industrial targeting. They require regulatory processes to guide discretion and political coordination to manage domestic and international welfare impacts.

Obviously, the current "New Learning" does not back any blunt "rejection of standard numerical objectives and fiscal rules" (Boone, as cited in Milenio, 2021) or the wholesale dismissal of principles of frugality and precaution in keeping one's house in order. It does, however, support the need to review guidelines for fiscal policy, not necessarily to reduce the restrictiveness of debt or deficit standards, but to reconsider, or better, to remember some fundamentals. Summer's suggested use of an increased debt basically evokes the time-honoured "golden rule" of public finance, which requires governments to borrow only to invest and not to fund current spending. It could be argued that the EU's RRF reflects this thinking. Yet when one leaves the lofty heights of economic discourse to enter EU realpolitik, perspectives change as one confronts the tinkering of policy implementation and concerns for the legitimacy and legality of EU involvement.

## Brussels' recovery fund

In 2021 and for the first time in its history, the EU Commission has taken on large scale debt in order to distribute aid to member countries. The debt should be repaid – at least in part – from new taxes at the EU level, which, except for the new levy on non-recyclable plastic, have not been decided and will most likely take years to put in place. Meanwhile, the loans will have to be paid out of member states' remittances. Judging from bond spreads, investors seemed to have confidence in the facility's ability to fire up EU recuperation; yet, analysts asked whether countries were able to identify viable projects, possessed the necessary institutional infrastructure to handle ambitious investment strategies or had the economic resilience to deal with competitive impacts.

According to the European Court of Auditors, Italy and Spain, the two principal beneficiaries of the scheme, had historically poor absorption rates. Between 2014 and 2020, both countries had only been able to use 40% and 39% of their respective shares of the EU structural investment funds.

In 2021, Italy was to have €209 billion available, almost one-third of the entire recovery budget. But one month prior to the application deadline, the country had not managed to narrow down its list of over 50 undetailed project proposals, nor had it identified who had to supervise project implementation. The Italian financial newspaper *Il Sole 24 Ore* (Cerretelli, 2021) commented: "After the 2011 crisis Italy did not ask for EU help, but it did not carry out the reforms either. … This constant avoidance of reforms has made us into the country with the lowest growth rate in Europe for the last 30 years, with a per capita income by now lower than the EU and euro zone average. …Europe is willing to fund Italy's rescue and revitalization precisely because it knows that it cannot coexist forever with its third largest economy on the brink of the abyss of national debt and with no economic growth. However, this revitalization cannot be done without our tangible commitment."

Meanwhile, Madrid had passed a decree designed to help Spain overcome its difficulties absorbing and spending EU funds. But the measure aimed to help modernise public administration, promote public-private sector collaboration in many fragmented investments and spend more under "urgent contracting rules". Some observers asked whether this was the most effective way to spend the fund and wondered why grants had not been tied to specific and clear reform commitments. Others cautioned that there was a risk that the central government would squander the capital for income replacement, social provisions or high-profile projects of little economic relevance. But criticism was not limited to Italy or Spain alone.

The German government, for example, wanted to put 42.7% of its RRF funds into the country's climate policy and energy turnaround and a further 20.3% was to be made available for the digitalisation of the economy and infrastructure. But the country was said to be largely replacing national funds rather than financing new investments and, instead, to be focusing on providing state aid to German industry. Berlin was clearly taking advantage of the EU's temporary extension of state aid measures to compensate companies for direct damages sustained as a result of the pandemic. By March 2021, the Commission had authorised around 370 aid measures; in terms of value, around 52% concerned German petitions. For some, companies in member states with lower spending capacities were disadvantaged by this. And yet, it could be argued that Berlin focused on maintaining its viable industrial foundations while building new infrastructures; it used its institutional apparatus to make the most of a regulatory opportunity, and, according to calculations of the Bundesrechnungshof, the German Supreme Audit Institution, would anyway be paying €65 billion more into the Fund than it would take out (Neue Zürcher Zeitung, 2021).

In all political skirmishes, however, one fundamental concern had often been overlooked: How would Next Generation EU be dealt with in the Union's fiscal framework and with what implications for the various member countries? In its December 2020 monthly report, the Bundesbank called for the EU debt to be counted towards national debt and deficits, just like the obligations arising from the European Financial Stability Facility, which are attributed to the EU countries. But, for several reasons, observers noted that the EU Commission was unlikely to agree to this. According to Darvas and Wolff (2021), "the repayment of EU debt cannot be clearly allocated to any national treasury. It is impossible to make reasonable estimates of how much each EU country would contribute to the repayment of the EU debt, starting from 2027 and running to 2058,…partly because it depends on many unknown future developments…in the next four decades. Moreover, some of the money for repayment is supposed to be raised from newly created EU taxes. …Allocating a 'federal' debt to national budgets would make that debt *de-facto* national debt. While the underlying taxpayers obviously are all EU tax subjects, the character of NGEU debt is clearly very different to national debt."

This reasoning is far from clear, but its implications are obvious. For one, member countries were agreeing to debts without understanding the repayment terms. Next, the Commission was set to create its own tax base. Finally, not considering NGEU financial expenditures as national debt allows them to be excluded from national structural balances and thus permits countries to avoid reducing their non-NGEU spending once the currently suspended fiscal rules are re-activated. Simulations of the latter show that this treatment would substantially reduce budget consolidation pressures in Italy, Spain, Poland and France from 2022 to 2024, while, and somewhat counter-intuitively, the large net-payer countries into NGEU, including Germany and the Netherlands, will have substantially larger fiscal adjustment needs under the EU fiscal rules. Therefore, taking NGEU expenditure outside of national debt "is a meaningful way of supporting the EU's fiscal stance. If it becomes a permanent feature of the EU's architecture, it would help with the recovery phase when fiscal rules start to constrain national fiscal policymaking. Moreover, it is an instrument to support the EU's major investment needs" (Darvas and Wolff, 2021).

The idea of a permanent EU budget has been supported by an astounding alliance made up of representatives of Greenpeace, European trade union leaders, investor and fund manager George Soros and even Christine Lagarde, the President of the ECB, who, already in October 2020, had called for the recovery fund to be made a permanent tool. But it runs counter to the public commitment of Germany's Angela Merkel and the Netherlands' Mark Rutte, who immediately after the agreement on the reconstruction plan in July 2020 assured their electorates that the debt would remain a one-off event and by no means result in joining a debt union. Since then, the German skepticism had grown. In the words of Kay Scheller (2021), President of the German Bundesrechnungshof, "(t)he recovery fund constitutes a socialization of debts and responsibility. If member countries cannot or do not want to pay their debt, the rest will have to foot the bill".

Oddly, preventing the eurozone from turning into a debt zone was also the objective of spokespersons on the other extreme of the political spectrum – except their action plan was rather different. Representatives of the Greek Syriza party and the French Green, visibly supported by the French economist Thomas Piketty, called for the ECB to write off the sovereign debt it holds in exchange for the debtors' commitment to invest to the same extent in climate protection. Lagarde, representatives of the Commission and the French Ministry of Finance shunned the idea – for now; a report from the German Bundestag (Kaiser, 2021), however, offered a categorical response: A debt write-off by the ECB is incompatible with the prohibition of monetary state financing pursuant to Article 123 (1) Treaty on the Functioning of the European Union (TFEU); it violates EU law and constitutes a breach of EU Treaties. In support of this position, in March 2021, a lawsuit was filed at the German Constitutional Court, accusing the ECB of straying ever further into a fiscal rescue mission in an attempt to ensure the cohesion of the eurozone rather than focusing on its duty – to maintain price stability. In an earlier decision in May 2020, the Court already had found that the ECB's monetary policy was but a thinly veiled budget policy, side-stepping elected parliaments and leaving taxpayers stuck with surging liabilities without democratic consent. And yet, on 20 April 2021, Germany's Constitutional Court allowed the country's government to ratify the pandemic recovery fund while the suit against the programme was pending. The Court stated that the underlying case is not per se inadmissible or without merit, as it cannot be ruled out that the liability risks, the fund's duration and the parliament's limited involvement may violate the constitution. But "delaying or derailing the package" was seen to "cause the EU to fall even further behind other advanced economies" (Matussek and Look, 2021). This, frankly, is an awkward argument.

As the political group that had filed the case continues to challenge the ruling, the current charge is likely to be extended to find that the financing of NGEU amounted to an unconstitutional landgrab by the Commission attempting to use the pandemic to broaden its remit and create its own, unwarranted fiscal foundation. It is unlikely that members of the "frugal" north will agree to illicit treaty changes – as a matter of principle; but even less so, as a matter of economic precaution. It can only be hoped that the Court returns to judging matters based on principles rather than expedience.

## Conclusion

By April 2021, the crisis in Europe had reached a magnitude that, in the eyes of some observers, either deepened lingering divides and threatened the EU's very existence, or, conversely, by forcing the Union to address the fundamental flaws of the euro area, provided an opportunity to reboot. In view of current proposals, chances for the latter are rather limited.

From the outset, the EU had to confront fundamental challenges faced by any union, political or otherwise. Alliances are viable only to the extent that they create and maintain procedural and substantive consensus and deference to it. They tend to expand to some point of saturation and disintegrate once membership grows further in numbers and diversity, its leadership is closed to participation or does not deliver benefits, and necessary reforms are delayed or not pursued by all (Doughtery and Pfalzgraph, 1984).

While European coordination is needed, it should be limited to areas where global markets are distorted, economies are beyond national/regional scale, and there is limited ability to upload local/national regulation as global standards. Decentralised coordination is best as it improves the quality of policy, economic efficiency and civic virtues.

Already in 2005, the EU failed to re-engineer its operations and gain legitimacy. Brussels tried to regain acceptance with a proposed constitutional reform but there was no review of the economics of governmental activities, no intention to set up a system that would enforce regulatory competition and use market principles to hold national governments accountable. The results are obvious and today, in 2021, it is likely that the limits have been reached. A debt union cannot provide the answer to Europe's call for shared adjustment. Economic reform, accountability and enforcement of constitutional commitments can.

## References

[CR1] Blanchard O (2019). Public debt and low interest rates. American Economic Review.

[CR2] Boscheck R (2006). EU Constitutional Governance: Failure as Opportunity?. Intereconomics.

[CR3] Cerretelli, A. (2021, 5 January), The European Obligations That Italy Still Fails To See, *Il Sole-24 Ore*, republished by BBC Monitoring Europe, 14 January.

[CR4] Darvas, Z. and G. B. Wolff (2021, 4 March), The EU’s fiscal stance, its recovery fund, and how they relate to the fiscal rules, *Bruegel Blog*.

[CR5] Doughtery, R. and J. Pfalzgraph (1984), *Theories of International Relations*, Harcourt.

[CR6] European Commission (2020a, 27 May), Europe’s moment: Repair and prepare for the next generation, Press release.

[CR7] European Commission (2020b), Identifying Europe’s recovery needs, *Commission Staff Working Document*, 98 final.

[CR8] Gros, D. (2020a), Europe and the COVID-19 crisis: the challenges ahead, *CEPS Policy Insights*, 2020–20.

[CR9] Gros D (2020). Lessons From the COVID-19 Crisis for Euro Area Fiscal Rules. Intereconomics.

[CR10] Hansen A (1934). Capital Goods and the Restoration of Purchasing Power. Proceedings of the Academy of Political Science.

[CR11] Hemerijck A C, Vandenbroucke F (2012). Social Investment and the Euro Crisis. Intereconomics.

[CR12] Hood, C. (1993), *Explaining Economic Policy Reversals*, Open University Press.

[CR13] Kaiser, T. (2021, 15 February), Warning from Germany, *Die Welt*, (translated into English), 10.

[CR14] Keynes J M (1936). The General Theory of Employment, Interest and Money.

[CR15] Krugman, P. (2020, 9 September), The case for permanent stimulus, *VoxEU*.

[CR16] Matussek, K. and C. Look (2021, 21 April), German Court Clears Road for EU Recovery Fund Ratification, *Bloomberg*.

[CR17] Milenio (2021, 11 January), Los países deben abrazar los objetivos, de la sostenibilidad a largo plazo: OCDE.

[CR18] Monastiriotis V (2013). A Very Greek Crisis. Intereconomics.

[CR19] Mundell R (1961). A Theory of Optimum Currency Areas. American Economic Review.

[CR20] Neue Zürcher Zeitung (2016, 11 March), Teuerung um jeden Preis.

[CR21] Neue Zürcher Zeitung (2021, 11 March), EU-Corona-Fonds: Der deutsche Rechnungshof sieht hohe Risiken für Bundeshaushalt.

[CR22] Rachel, l. and T. D. Smith (2015), Secular drivers of the global real interest rate, *Bank of England Working Papers*, 571.

[CR23] Scheller, K. (2021, 23 March), quoted in: Streit um die Corona-Milliarden, *Berliner Morgenpost*, 6.

[CR24] Summers L H (2014). U.S. Economic Prospects: Secular Stagnation, Hysteresis and the Zero Lower Bound. Business Economics.

[CR25] Summers L H (2016). The Age of Secular Stagnation. Foreign Affairs.

[CR26] Truger A (2020). Reforming EU Fiscal Rules: More Leeway, Investment Orientation and Democratic Coordination. Intereconomics.

[CR27] Wicksell, K. (1898), *Geldzins und Güterpreise: Eine Studie über den Tauschwert des Geldes bestimmenden Ursachen*, Fischer.

